# Protein–Protein Interactions Mediated by Intrinsically Disordered Protein Regions Are Enriched in Missense Mutations

**DOI:** 10.3390/biom10081097

**Published:** 2020-07-24

**Authors:** Eric T. C. Wong, Victor So, Mike Guron, Erich R. Kuechler, Nawar Malhis, Jennifer M. Bui, Jörg Gsponer

**Affiliations:** Michael Smith Laboratories, University of British Columbia, Vancouver, BC V6T 1Z4, Canada; eric_wong@live.ca (E.T.C.W.); victor.kaiming.so@gmail.com (V.S.); mike_guron@alumni.ubc.ca (M.G.); erich.kuechler@gmail.com (E.R.K.); nmalhis@msl.ubc.ca (N.M.); jennbui@gmail.com (J.M.B.)

**Keywords:** intrinsically disordered proteins, single nucleotide variants, protein–protein interactions, interface core and rim, human disease

## Abstract

Because proteins are fundamental to most biological processes, many genetic diseases can be traced back to single nucleotide variants (SNVs) that cause changes in protein sequences. However, not all SNVs that result in amino acid substitutions cause disease as each residue is under different structural and functional constraints. Influential studies have shown that protein–protein interaction interfaces are enriched in disease-associated SNVs and depleted in SNVs that are common in the general population. These studies focus primarily on folded (globular) protein domains and overlook the prevalent class of protein interactions mediated by intrinsically disordered regions (IDRs). Therefore, we investigated the enrichment patterns of missense mutation-causing SNVs that are associated with disease and cancer, as well as those present in the healthy population, in structures of IDR-mediated interactions with comparisons to classical globular interactions. When comparing the different categories of interaction interfaces, division of the interface regions into solvent-exposed rim residues and buried core residues reveal distinctive enrichment patterns for the various types of missense mutations. Most notably, we demonstrate a strong enrichment at the interface core of interacting IDRs in disease mutations and its depletion in neutral ones, which supports the view that the disruption of IDR interactions is a mechanism underlying many diseases. Intriguingly, we also found an asymmetry across the IDR interaction interface in the enrichment of certain missense mutation types, which may hint at an increased variant tolerance and urges further investigations of IDR interactions.

## 1. Introduction

Driven by the goal of understanding genetic diversity in the human population and how this diversity affects disease likelihood, efforts in sequencing human genomes have provided vast amounts of sequence variants, also known as single nucleotide variants (SNVs). If SNVs are located in protein-coding regions of the genome and are non-synonymous, they can result in premature stop codons (i.e., nonsense mutations) or substitutions of amino acids (i.e., missense mutations), both of which could impact the biological function of the encoded proteins. As a result, SNVs can be categorized as deleterious, benign, or even beneficial for human health. Studies have shown correlations between the phenotypic effects of SNVs and their localization to different functional regions of proteins [1,2,3,4].

Protein structural data is critical for mapping SNVs to functional regions and for understanding the molecular mechanism through which they lead to functional alterations. Each residue contributes differently to protein folding and function, and the different constraints on the residues are mirrored in the localization patterns of the SNVs observed in the protein structure. This relationship first became evident when disease-associated SNVs, specifically those that cause missense mutations, were mapped onto protein structures [5,6]. Mutations associated with diseases were shown to be enriched at active sites and buried regions that provide structural stability [2,7]. Disease-associated missense mutations were also found enriched at protein–protein interaction (PPI) interface regions, which does not come as a surprise given that the majority of proteins require interactions with other proteins to perform their functions properly. Importantly, the enrichment of disease-associated mutations at the protein interface, relative to the non-interface surface, shows much greater contrast when focusing on the residues at the core of the interface [2]. PPI interfaces can be divided into core and rim regions using the protein complex structures, where the core residues become buried upon binding while the rim residues remain relatively solvent-exposed and typically form the perimeter of the interface [8,9]. Disease-associated missense mutations are more common in the interface core, especially at the hotspot residues that contribute most to protein interaction affinity [2].

The enrichment of disease-associated missense mutations at interface regions clearly suggests that the disruption of PPIs is likely a common mechanism for altered biological function and disease [10]. An extensive mutagenesis study provides strong support for this hypothesis [1]. This study revealed that the majority of the tested disease-associated mutations disrupted PPIs, and the interaction-disrupting mutations can be divided into quasi-null and edgetic mutations. A quasi-null mutation abolishes all of a protein’s interactions, likely through destabilization of the protein, while an edgetic mutation removes a specific subset of interactions. These edgetic mutations are more often found in interface regions, altering their binding properties. Furthermore, disease-associated mutations in different interface regions of the same protein can lead to different diseases, providing an explanation for the pleiotropic effects of disease-associated genes [10].

Structural mapping has also been exploited to investigate the functional impact of SNVs associated with specific classes of diseases, most prominently cancer. Somatic SNVs associated with cancer are of particular interest because some of them drive the propagation of the tumor cells, which contrasts the broader disruptiveness of germline disease-associated SNVs [6,11]. Somatic cancer-associated SNVs from tumor tissues were also found enriched at functional regions, but studies have reported higher enrichment at the protein surface compared to the buried regions [6,11]. Importantly, the properties of the SNVs depend on the native and cancer-associated functions of the proteins. Cancer-associated SNVs in oncoproteins cause gain-of-function and are more commonly found on the surfaces of proteins as well as clustered and recurrent in specific sequence positions [4,12,13]. While SNVs that can activate oncoproteins are limited to a select few residues, these oncoprotein SNVs appear to be under stronger positive selection in tumor cells, highlighting their key roles in driving oncogenesis [14]. Cancer-associated SNVs in tumor suppressor proteins are found enriched at the buried regions and are more often scattered across the sequence, resulting in destabilizing effects similar to the typical germline disease-associated SNV [4].

Recent sequencing efforts have also enabled the identification of SNVs present in the healthy human population. These SNVs and their observed frequency, as annotated in databases such as gnomAD [15], provide a first glimpse of the natural sequence variations in human populations. They are not directly associated with diseases and are depleted from functionally critical protein regions such as protein interfaces while their enrichment is inversely correlated with evolutionary sequence conservation [2,16], sharply contrasting the trends seen in disease-associated SNVs. This finding is unsurprising as the tolerance for sequence variation is likely to be higher at non-functional, less-conserved protein regions.

Studies of SNVs from the healthy population often make a distinction between common and rare variants. Common SNVs, which are often defined as those with greater than 1% frequency in the population, are typically functionally neutral [17,18]. Some common SNVs may even provide a selective advantage and may be beneficial for the adaptation of the population to environmental changes or stressors [19,20]. The localization patterns of these common SNVs most strongly contrast with disease-associated SNVs. On the other hand, rare SNVs account for the majority of variants in the population [21]. Rare SNVs consist of mutations under negative selection as well as novel mutations and thus are enriched in deleterious mutations [22,23,24]. These mutations also tend to have greater effects on function, which was demonstrated by a study on human height distribution that found an inverse correlation between frequency and phenotypic effect [25]. Therefore, common and rare SNV datasets contain SNVs with low and medium levels of deleteriousness on average, which provides contrast with the highly deleterious disease-associated SNVs.

Structural analyses of SNVs, most frequently on the SNVs causing missense mutations, have previously focused on independently-folding (i.e., globular) domains. However, many PPIs are mediated by protein regions that are not confined in a single folded conformation prior to binding, namely intrinsically disordered regions (IDRs) that participate in PPIs (interacting IDRs) [26,27,28]. IDRs are underrepresented in interaction and structural datasets [28,29,30], but IDRs are increasingly recognized for their prevalence and their critical roles in regulatory intermolecular interactions [31]. It has been hypothesized that some traits make IDRs particularly suitable for interactions involved in signaling and regulation [31], complementing globular domains that more often perform catalytic functions. IDRs contribute large interaction surfaces in the form of compact interaction modules such as shorter peptide motifs and longer molecular recognition features (MoRFs) [26,28,32]. It has been estimated that IDRs in the human proteome contain ~132,000 binding motifs [28]. Peptide motifs and MoRFs can be used in combinatorial ways due to alternative splicing and the modulation of their interaction propensities via post-translational modifications [33,34]. Moreover, the flexibility of IDRs allows multivalent and fuzzy, often promiscuous interactions with multiple partners as well as fast binding kinetics and low-affinity high-specificity partnerships [35,36,37]. Given these traits, it is not surprising that IDRs are a common feature of hubs in PPIs, which are proteins that make the largest number of interactions and thus greatly influence the connectivity of PPI networks [38,39,40].

Given the significance of IDR-mediated protein interactions, it is pertinent to know whether disease, common, and rare SNVs are similarly enriched/depleted at the interfaces of IDR-mediated interactions (IDR interactions) as at interfaces between folded domains (globular interactions). It has been established that IDRs are generally less enriched in disease SNVs compared to other protein regions and that they exhibit higher evolutionary rates that could be attributed to weaker structural constraints [41,42]. Nonetheless, with an estimated 22% of disease SNVs located in IDRs and a higher concentration of these mutations in IDRs that are involved in PPIs, the importance of understanding the mutations in IDRs should not be understated [43]. A study of sequence motifs revealed enrichment of disease-associated SNVs compared to benign SNVs [44], suggesting that the function-disrupting substitutions are concentrated in the interaction-mediating elements residing in IDRs. In the light of this finding and the fact that IDRs interact mostly with folded domains, although IDR–IDR interactions have been reported, one could expect the interfaces of the globular partners of IDRs (IDR-partners) to exhibit the familiar trends in SNV enrichment/depletion that have been observed in globular interaction interfaces. However, IDR interactions and globular interactions exhibit differences in both structure and function [27,45,46,47,48], and we have previously found IDR-partner interfaces to have distinctive physicochemical and geometric properties [49]. Moreover, IDR-partner interfaces bind to inherently dynamic IDRs that are potentially more accommodating to changes in the interface. Therefore, the IDR-partner interfaces may exhibit distinctive mutation enrichment patterns, which demand a closer inspection.

**Figure 1:**
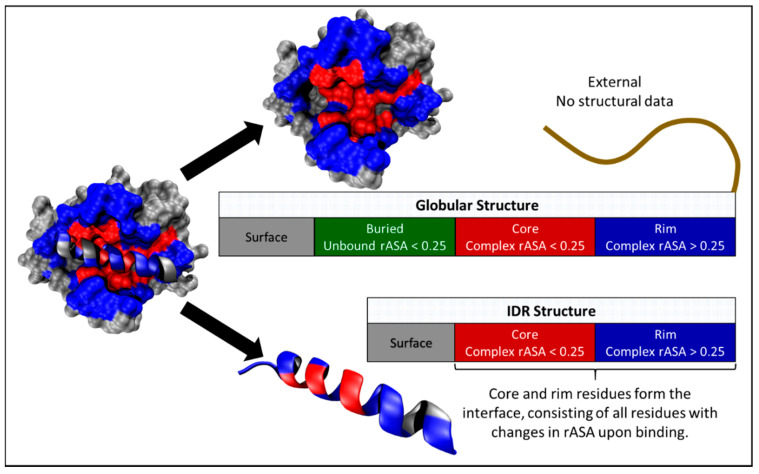
Structural regions analyzed in this study. The structural regions were defined based on solvent-accessible surface areas measured from protein complex structures. Residues with changes in relative solvent accessible surface area (rASA; see Methods) between the bound and unbound conformations were defined as core residues (red) if rASAs are smaller than 0.25 and rim residues (blue) if rASAs are greater than 0.25 in the bound structures. Buried residues are non-interface residues with rASAs smaller than 0.25 in the unbound structures, and the remainder are surface residues (gray). Because the full-length protein often contains regions without structural data coverage, these structurally undefined sequences were classified as external regions in our analyses.

In this work, we built on previous studies analyzing the localization of several categories of missense mutations in protein complex structures, but we focused on IDR interactions. Specifically, we analyzed the bias of missense mutations among interface residues as well as buried and surface non-interface residues (Figure 1). Importantly, we separated protein interfaces into core and rim regions because we and others have demonstrated characteristic differences in the two interface regions, including residue composition and SNV enrichment [2,9,46,49]. To calculate mutation enrichments, we mapped disease-associated SNVs from SwissVar, somatic cancer-associated SNVs from COSMIC, and SNVs from gnomAD that cause missense mutations onto available structures of IDR interactions, as well as globular interactions which serve as a control [15,50,51]. Our analyses reveal that interface regions of interacting IDRs are at least as enriched in disease-associated SNVs as globular interactions and exhibit depletion of gnomAD SNVs, especially at the interface core regions. Notably, IDR-partner interfaces exhibit a strong presence of disease-associated SNVs. However, our analyses may also provide preliminary evidence of a greater tolerance for common gnomAD SNVs at IDR-partner interfaces, which deserves further investigation. Overall, our findings are concordant with studies that have associated IDRs with numerous diseases, especially cancer [52,53,54,55].

## 2. Materials and Methods

### 2.1. Structural Data

The structural data of protein interactions consists of human proteins downloaded from the RCSB Protein Data Bank (PDB) in September 2018 (http://www.rcsb.org/). For each structure, the model of the biological unit was selected whenever available, and the first model was used when the PDB file contains multiple models. Complex structures that only consist of carbon-alpha coordinates or are too large for the computational software we utilized were removed. Protein interactions were analyzed pairwise by iterating through all pairs of protein chains in each PDB file, focusing only on human heteromeric interactions and removing pairs with no physical interaction, which was determined through calculating changes in solvent accessibility. FreeSASA was used for calculation of solvent accessible surface area (SASA) of the residues of each protein chain in their bound and unbound states [56], where the unbound state was the structure of each protein chain in isolation. Physically interacting protein chain pairs are those with a change in total SASA between their bound and unbound states.

For each interaction pair, relative solvent accessible surface area (rASA) of protein residues were calculated to categorize the residues into protein regions. The SASA of each residue of the protein structures was normalized by the SASA of the residue type “X” calculated in a Gly-X-Gly peptide in extended conformation [9]. The residues were placed into structural categories based on their rASA in their bound and unbound states [9]. Surface and buried regions consist of residues above and below 0.25 rASA in the isolated protein chain, respectively. All residues with a change in rASA between the bound and unbound states were defined as interface residues. The interaction interface consists of the rim residues, which have rASA > 0.25 in the bound state, and the core residues, which have rASA < 0.25 in the bound state. Subsequently, the categorized residues were mapped to UniProt sequences [57]. Supplementary Figure S1 provides an overview of the construction process of the interaction structure datasets, as well as the number of structures and proteins involved. The dataset of globular interactions encompasses all interaction pairs, which is justifiable since the dataset of IDR interactions is small in comparison.

### 2.2. Defining Intrinsically Disordered Regions (IDR) Interaction Datasets

IDR interactions were identified by mapping IDRs onto UniProt sequences and subsequently screening PDB complex structures for the IDRs. A curated dataset of IDRs was extracted from the MobiDB database in September 2018 [58]. The MobiDB database contains protein regions annotated as curated linear interacting peptides (LIPs), which consist of IDRs aggregated from multiple databases. For this study, entries from the ELM were excluded since they contain short linear motifs (SLiMs) that are found not only in disordered regions but also in globular regions [59].

For each UniProt sequence with IDRs defined by MobiDB, we iterated through all interaction pair structures to identify all instances of the IDRs. For an interaction pair structure to be labeled as an IDR interaction, one of the protein chains must overlap with an IDR sequence. A protein chain was labeled as an interacting IDR if more than 50% of the interface residues were within an IDR defined in MobiDB. Furthermore, protein chains with more than nine buried residues in their unbound states were excluded from interacting IDRs, thereby removing chains that potentially contain independently-folding regions. Once all the interacting IDR and IDR-partner interaction pair structures were defined, the remaining structures were excluded from the IDR interaction dataset.

### 2.3. Mapping Mutations to Globular and IDR Interaction Structural Data

SNVs in protein-coding regions that cause missense mutations and are associated with diseases were sourced from the SwissVar and COSMIC databases. The SwissVar SNV dataset consists of disease-related germline mutations [50]. The COSMIC SNV dataset consists of curated cancer mutations, excluding mutations annotated with genome-wide screens and single nucleotide polymorphisms [51]. The COSMIC database contains both cancer driver and passenger mutations, and the mutations may come from proteins that are labeled as oncoproteins or tumor suppressor as well as belonging to neither or both of those categories. Because of the functional differences between oncoproteins and tumor suppressors, we further divided the COSMIC-SNV-mapped proteins into those labeled exclusively as oncoproteins or tumor suppressors by using the datasets constructed by Brown et al. [14].

SNVs that cause missense mutations and are not associated with diseases, and thus are generally considered benign, were sourced from the gnomAD database [15]. The gnomAD SNV dataset consists of SNVs from the healthy human population as well as their frequencies, allowing their categorization into common and rare SNVs. We analyzed SNVs with a frequency between 0.1 and 10^−6^. As the amount of common SNV data is very small, we used a comparatively relaxed threshold frequency of 0.001 to define our high-frequency SNV dataset, which provides a subset of SNVs with a greater fraction of benign mutations for analysis. For comparison, we also analyzed a set of rare SNVs that have frequencies between 5 * 10^−6^ and 10^−6^.

We merged mutation data with structural data by mapping all missense mutations to the interaction pair structures through their shared UniProt sequences [57]. We subsequently iterated through all the interaction pair structures and merged all the structural and mutation data for each UniProt sequence. Merging the SwissVar SNV, COSMIC SNV, oncoprotein SNV, tumor suppressor SNV [14,51], and gnomAD SNV datasets with the globular, MobiDB interacting IDR [58], and IDR-partner datasets resulted in 15 combined datasets. For each of the 15 combined datasets, UniProt sequences lacking either structural data or mutations were removed. In case of overlap between multiple PDB structures, the residue structural label was decided by their priority from highest to lowest: core, rim, buried, surface, and unstructured (external region; see Figure 1). In other words, if a protein residue position was an interface core residue in one structure and a non-interface surface residue in another, the residue will be labeled as an interface core residue in the merged data. In the case of the IDR interaction dataset, the UniProt residues were also labeled as interacting IDR or IDR-partner. The tabulated residues and mutations for all datasets are presented in Supplementary Table S1.

### 2.4. Odds Ratio Calculations

We used odds ratios (ORs) to compare mutation enrichment between protein regions, as described previously by David and Sternberg [2]. OR values higher than one denote enrichment of missense mutations at the specified regions, while depletion results in values smaller than one. The probability of mutation (*p*) in region *i* was given by the number of mutated positions (m) in region *i* divided by the number of residues (r) in region *i*, i.e.,:

(1) $$p_{i}=m_{i}/r_{i}$$

The odds ratio of mutations in region *i* over *j* is:

(2) $$OR_{ij}=\frac{\left(p_{i}/\left(1-p_{i}\right)\right)}{\left(p_{j}/\left(1-p_{j}\right)\right)}$$

The standard error for the natural log of the odds ratio is [3]:

(3) $$SE\_LOR_{ij}=\sqrt{\frac{1}{m_{i}}+\frac{1}{r_{i}-m_{i}}+\frac{1}{m_{j}}+\frac{1}{r_{j}-m_{j}}}$$

The standard error for the natural log of the odds ratio was used to estimate the standard error of the odds ratio [10]:

(4) $$SE\_OR_{ij}≅OR_{ij}*SE\_LOR_{ij}$$

The standard error of the odds ratio was used to define the error bars in the bar plots of ORs, which were generated using the ggplot2 module in R [60]. The *p*-values of ORs were calculated using the chi-square test in R and are reported in Supplementary Table S2. The enrichment of mutations at each protein region was determined using the full-length protein as the reference, i.e., region $r_{j}$ is the total number of residues in a dataset. Therefore, an OR of the interacting IDR dataset would be calculated with $p_{j}$ equal to the number of mutations in all proteins containing interacting IDRs divided by the total length of those proteins. Correspondingly, a $p_{j}$ of the IDR-partner dataset would be calculated based on the subset of proteins containing IDR-partner structures.

## 3. Results

### 3.1. Disease-Associated Single Nucleotide Variants (SNVs) Are Enriched at IDR Interaction Interfaces

To pursue our goal of revealing whether IDR interfaces exhibit familiar trends in SNV enrichment/depletion that have been observed in globular interaction interfaces, we first repeated the enrichment analysis for globular interactions and set baselines for the comparisons with IDR interactions. We collected structures of heteromeric protein complexes from the Protein Data Bank (PDB) to assemble the globular dataset (see Methods for details). We divided residues in these complexes into structural regions based on their solvent accessibility in bound and unbound states (see Methods for details). Briefly, the surface and buried regions were defined as residues that are exposed and unexposed to solvent in the unbound state, respectively. The interface region was defined as residues that become more buried upon complex formation, i.e., residues that change in solvent exposure when comparing the complex to the separate protein chains (Figure 1). We further divided the interface region into core and rim, which are the central and peripheral sections of the interface, respectively, because of the differences in sequence and structural characteristics between the two regions [2,9].

**Figure 2:**
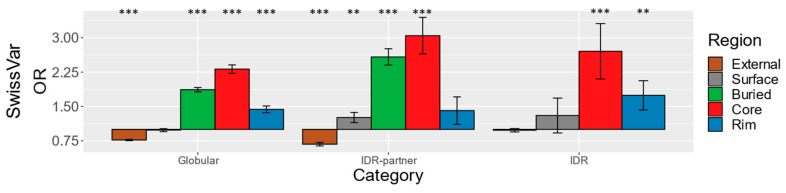
Odds ratios of SwissVar single nucleotide variants (SNVs). An odds ratio (OR) is calculated for each protein region using all residues in the dataset as the reference distribution. The bar graph plots the ORs (*Y*-axis) of each protein category and protein region (*X*-axis). Each OR is the odds of mutation in the specific region divided by the odds of the full-length parent proteins. The *Y*-axis is centered at one, and ORs > 1 show enrichment while ORs < 1 show depletion. Structural regions are color-coded (see Figure 1). Statistical significance is denoted by asterisks: * *p*-value < 0.05, ** *p*-value < 0.01, *** *p*-value < 0.001. ORs and *p*-values can be found in Supplementary Table S2.

We began our comparison of enrichments by analyzing the distribution of disease-associated missense mutations from the SwissVar database (SwissVar SNVs) in the globular dataset. After mapping the mutations to the structural regions, we calculated the enrichment/depletion of mutations at each structural region using odds ratios (ORs) [2], with OR > 1 indicating enrichment of mutations relative to the full sequence distribution (see Methods for details). Our globular interaction dataset shows the highest enrichment of SwissVar SNVs at the buried and interface core regions of proteins (buried OR = 1.9, *p*-value ≤ 10^−99^; core OR = 2.3, *p*-value ≤ 10^−99^; Figure 2). Buried residues of globular domains are typically more critical to the structure and stability of the protein, while core residues tend to contribute strongly to protein binding, so the substitution of these residues will more likely disrupt function. Thus, these enrichment patterns are consistent with the disease association of the mutations. These observations are also in agreement with previous studies of disease-associated missense mutations that reported the strongest mutation enrichment at the buried and core regions [1,2,61]. Although David et al. reported more significant enrichment at the buried region, this discrepancy could be explained by differences in rASA thresholds used in defining structural regions [2]. The rim region has an OR of 1.4 (*p*-value = 2.5 * 10^−12^), which is much lower than the interface core region but still suggests stronger functional constraints than the non-interface surface region, which has an OR of 1.0 (Figure 2).

Next, we repeated this analysis for IDR interactions. We identified these interactions, i.e., interactions between IDRs and folded IDR-partners, by mapping curated IDRs from the MobiDB database to PDB complex structures (see Methods for details). Notably, because PDB structures are often limited to crystallizable protein complexes, the IDRs in our datasets are generally regions that fold upon binding, such as MoRFs and peptide binding motifs (see Results Section 3.3 and Discussion). We then calculated ORs for the different protein regions, as it was done for the globular interactions, calculating the denominator odds using the number of mutations and sequence length of proteins that contain either IDR or IDR-partner structures (see Methods for details). IDR-partners in this dataset are independently folded domains while IDRs, by definition, are not, so we calculated the enrichments for IDR-partners and interacting IDRs separately (Figure 2 middle and right). For the IDR-partner, the OR calculations reveal a similar picture of enrichment as for the globular interactions. Specifically, SwissVar diseases-associated mutations are found enriched at the buried parts as well as the interaction interface consisting of core and rim residues. The enrichment of SwissVar mutations at the IDR-partner interface core region as well as its buried parts are even more pronounced than at the globular interaction regions (buried OR = 2.6, *p*-value = 3.3 * 10^−45^; core OR = 3.0 *p*-value = 7.3 * 10^−19^). The IDRs themselves also show enrichment of SwissVar disease-associated mutations at interface locations (Figure 2 right). These mutations are found significantly enriched at both the core and the rim regions of the interface, but the enrichment is particularly pronounced at the interface core (core OR = 2.7, *p*-value = 4.3 * 10^−6^; rim OR = 1.7, *p*-value = 2.4 * 10^−3^). It needs to be noted that the IDRs lack buried residues because they predominantly interact by adopting secondary but not tertiary structures. This analysis of mutations from SwissVar clearly demonstrates that disease missense mutations are not only found enriched at the core of classical interfaces between folded domains but also at the core of interfaces between IDRs and their partners. This result may suggest that the interface core of interacting IDRs and IDR-partners have functional roles that are very susceptible to disruption by amino acid substitutions, maybe as susceptible as the core of globular interfaces.

**Figure 3:**
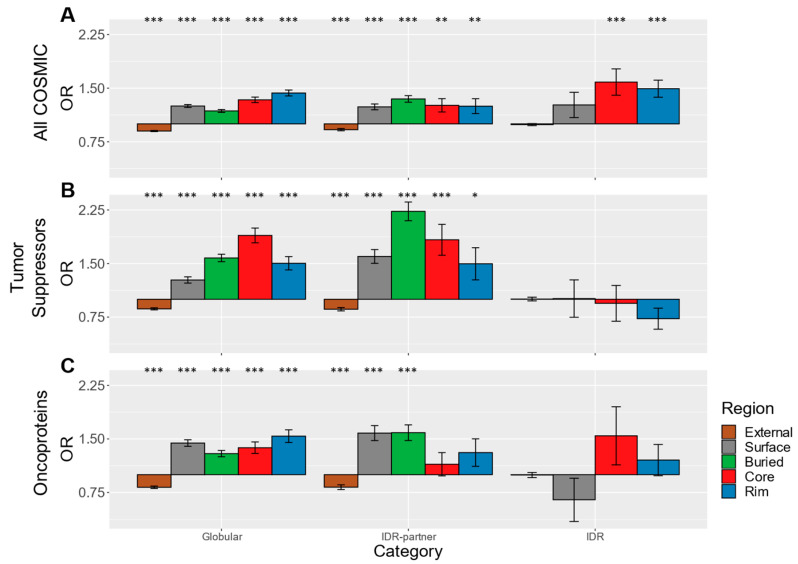
Odds ratios of COSMIC SNVs. (**A**) A bar graph of odds ratios of all COSMIC SNVs. The odds ratios of the subsets of proteins that were categorized as (**B**) tumor suppressors and (**C**) oncoproteins, respectively. See Figure 2 for details. *p*-values for all odds ratios can be found in Supplementary Table S2.

Compared to germline SwissVar mutations, somatic cancer-associated mutations from the COSMIC database are known to have different enrichment patterns and mechanistic properties, which prompted us to analyze them independently. Past studies have shown a distinctively greater tendency for cancer-associated mutations to occur in protein surface and interface regions [6,11], in contrast to the disease-associated germline mutations that favor the buried region [2]. Using the same procedures for mapping mutations to structural data and evaluating mutation enrichment, we found that the COSMIC cancer-associated missense mutations also exhibit enrichment at functional regions of the globular and IDR interaction sets (Figure 3A), although to a lesser degree than SwissVar SNVs. Indeed, ORs closer to one, specifically in the globular interaction proteins and in the IDR-partners, indicate weaker enrichment patterns compared to SwissVar SNVs. This difference could be attributed, at least in part, to the presence of passenger mutations in the COSMIC SNV dataset, which are missense mutations identified in cancer tissue that do not contribute to tumor growth and are under weak negative or no selective pressure [62]; therefore, they are expected to have a more uniform distribution across protein regions. Notably, the enrichment at the globular buried region is significant but relatively weak (Table S2), which is consistent with previous observations [6,11]. Interestingly, the rim regions of both the globular interactions and the IDR-partners show significant enrichment levels equal to those of the core regions. This finding contrasts the observations for SwissVar SNVs (Figure 2) and is intriguing since rim residues tend to contribute less to binding affinity when compared to the core. Most importantly, the highest ORs are observed in the interacting IDR core and rim (core OR = 1.6, *p*-value = 9.6 * 10^−5^; rim 1.5, *p*-value = 8.9 * 10^−7^). This enrichment at interacting residues of the IDRs contrasts the known depletion of COSMIC SNVs within IDRs in general [41,63], emphasizing a strong association between cancer and interacting IDRs and, more specifically, their interface core residues.

Cancer development and progression are generally driven by the inactivation of tumor suppressors and the activation of oncoproteins. Therefore, selective pressures that act on tumor suppressors and oncoproteins in cancer cells may generate a distribution of missense mutations that reflects more closely the functional importance of the affected residues. While we mapped COSMIC missense mutations across many proteins for our analysis, only small subsets of these proteins are verified as tumor suppressors and oncoproteins that drive oncogenesis [13]. As we were interested in the mutation distribution difference between tumor suppressors and oncoproteins, we repeated the analysis while selecting only proteins that were labeled as tumor suppressors and oncoproteins, and we excluded proteins that were annotated in both categories to segregate them and study their differences, as it was done by Brown et al. [14]. As we expected, missense mutations in tumor suppressors show enrichment patterns reminiscent of the SwissVar SNVs, in both the globular interactions as well as IDR-partners (Figure 3B). Most prominent is the statistically significant enrichment of mutations at buried and interface core regions. This result is consistent with previous reports of cancer-associated missense mutation enrichment at the buried region of tumor suppressors [12] and an expected loss of function when mutations hit buried residues important for protein stability. In contrast, the enrichment patterns for globular and IDR-partner oncoproteins (Figure 3C) more resemble patterns observed in the full COSMIC SNV dataset (Figure 3A). Interestingly though, the core regions of IDR-partners that are oncoproteins are not statistically enriched in COSMIC missense mutations (core OR = 1.1, *p*-value = 0.4). Unfortunately, the numbers of cancer-associated missense mutations that map to interacting IDRs from tumor suppressors or oncoproteins are very small, too small for confident interpretation (i.e., all ORs are not statistically significant; see Table S2). Due to the limited data, which results in a lack of statistical significance, we can only speculate on the observed trends. Interacting IDR interface regions from tumor suppressors do not appear enriched in COSMIC missense mutations, which is consistent with the idea that mutations in an interacting IDR are less likely to lead to a loss of function of the protein compared to mutations in the buried regions of globular domains. By contrast, interacting IDR interface cores from oncoproteins appear enriched in cancer missense mutations, which mirrors the enrichment of cancer missense mutations observed in the IDR core of all analyzed proteins (Figure 3A) and implies that these IDR interactions hold functions in cancer-associated pathways.

### 3.2. gnomAD SNVs Are Depleted at IDR Interaction Interfaces

Finally, we analyzed missense mutations from gnomAD to investigate how mutations present in the general population are distributed across structural regions in globular, interacting IDR and IDR-partner proteins. The mutations from gnomAD (gnomAD SNV) are observed in a population of healthy individuals, so these missense mutations are typically not directly associated with diseases. David and Sternberg previously studied non-disease-associated missense mutations annotated in the UniProt database and showed that these variants are depleted from functionally critical protein regions [2]. Specifically, they revealed enrichment at the rim and surface regions and depletion at the buried and interface core regions. The gnomAD SNV data is from large-scale genome sequencing projects, which also allows the study of rare missense mutations that were previously not detectable. Thus, in addition to analyzing all gnomAD SNVs, we also analyzed subsets of gnomAD SNVs with frequencies from 5 * 10^−6^ to 10^−6^ (rare SNVs) and from 0.1 to 0.001 (high-frequency SNVs). Studies have suggested that some mutations with very low frequencies can have deleterious effects [23,64], so the high-frequency SNVs should more accurately reflect the localization of benign mutations.

**Figure 4:**
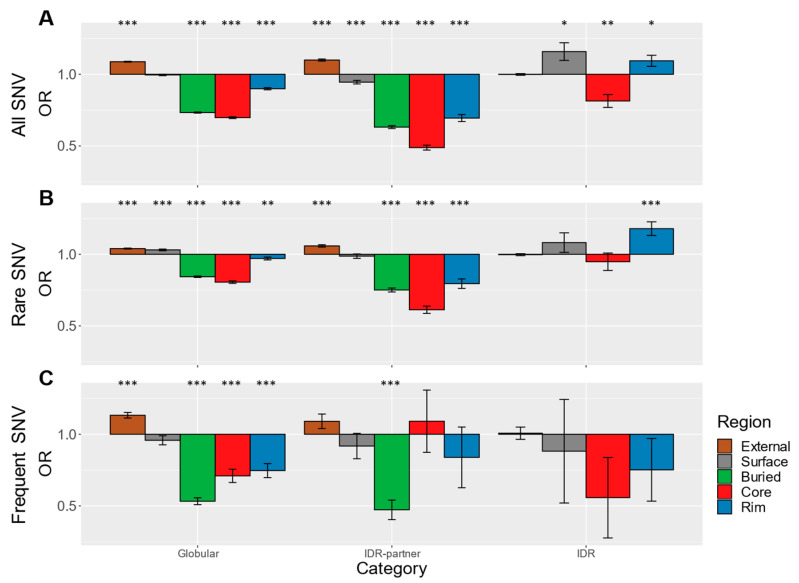
Odds ratios of gnomAD SNVs. (**A**) Odds ratios are calculated using gnomAD SNVs of frequencies between 0.1 to 10^−6^. (**B**) Odds ratios are calculated using gnomAD SNVs of frequencies between 5 * 10^−6^ to 10^−6^, i.e., rare SNVs. (**C**) Odds ratios are calculated using gnomAD SNVs of frequencies between 0.1 to 0.001, i.e., high-frequency SNVs. See Figure 2 for details.

We first present results from the full gnomAD SNV dataset (frequency 0.1 to 10^−6^) since it contains the largest number of mutations by far, thereby providing more reliable results. Results for the entire gnomAD SNV datasets were generated through the same procedures of mutation mapping and OR calculation. The ORs calculated for the globular dataset indicate that gnomAD SNVs causing missense mutations are significantly depleted at structured parts of proteins, especially at the functionally critical buried and interface core regions (Figure 4A). Compared to the ORs of the globular interaction set, the IDR-partner’s ORs indicate more substantial depletions of gnomAD missense mutations from functional regions. Particularly depleted of gnomAD mutations is the core region of IDR-partners (OR = 0.5, *p*-value = 3.0 * 10^−67^). This finding is contrasted by the relatively high and significant ORs of the surface and rim regions of interacting IDRs. However, the interface core of interacting IDRs is also depleted of gnomAD missense mutations (OR = 0.8, *p*-value = 2.1 * 10^−3^), and interacting IDRs as a whole are not enriched in these SNVs (i.e., surface, buried, core, and rim combined; OR = 1.0; Figure 4A). Together, these findings highlight, again, the functional importance of the core residues in both IDR-partners and interacting IDRs.

When we isolated the rare SNVs (frequency 5 * 10^−6^ to 10^−6^; Figure 4B), which contain variants that are generally seen only once in the available population sample, we still observed a significant depletion of the functional regions from missense mutations. This depletion is particularly pronounced for the IDR-partner core region (OR = 0.61, *p*-value = 2.8 * 10^−27^). However, the magnitude of the depletion is overall smaller than for all gnomAD SNVs. ORs closers to unity may be rationalized by the higher percentage of novel and deleterious SNVs among rare variants (see discussion). Interestingly, an exception to the weakening depletion pattern is the significant enrichment of rare SNVs at the interface rim of IDRs (OR = 1.2, *p*-value = 2.5 * 10^−4^). Similar to the result for all gnomAD SNVs (Figure 4A), the missense mutation enrichment outside the core of the interacting IDR is consistent with the previous observations of higher mutation rates for IDRs in general [65,66].

The relatively subdued enrichment patterns of the rare SNV missense mutations are juxtaposed by the high-frequency SNV dataset. We investigated the high-frequency SNVs (frequency 0.1 to 0.001; Figure 4C) because these mutations are the most likely to be benign based on their recurrence in healthy individuals. In the globular interaction dataset, the high-frequency SNVs that cause missense mutations are generally more strongly depleted from the functional regions compared to the complete gnomAD SNVs dataset (Figure 4C), which is consistent with the expected greater proportion of benign mutations in the high-frequency SNVs. For both globular interactions and IDR-partners, the buried region is the most devoid of high-frequency SNVs (globular OR = 0.53, *p*-value = 4.9 * 10^−46^; IDR-partner OR = 0.47, *p*-value = 7.7 * 10^−8^). Interestingly, compared with all gnomAD SNVs, the ORs of the high-frequency SNVs for interface core regions do not decrease proportionately with the buried regions. The divergence of trends in the buried and core regions is most striking for the IDR-partners, which have a relatively large proportion of high-frequency SNVs in the core region (OR = 1.1, *p*-value = 0.7). In contrast, the high-frequency SNVs appear relatively depleted from the interacting IDR core and rim regions, but results are inconclusive due to the scarcity of high-frequency SNVs with structural data for interacting IDRs (core OR = 0.56, *p*-value = 0.2; rim OR = 0.75, *p*-value = 0.3).

### 3.3. Robustness of Datasets and Findings

As some of the datasets in our analysis are small, it is possible that our study was influenced by an overrepresentation of a few specific domains. Therefore, we searched our interaction sets to test for overrepresented Pfam domains [67]. Supplementary Figure S2 shows the number of proteins containing the 20 most frequent Pfam domains in each interaction set and mutation data analyzed. This analysis clearly shows that the majority of the highest-ranked domains have similar numbers of occurrences, with a few exceptions. In the globular dataset (Figure S2A–C), the protein kinase domain (Pfam: PF00069) stands out with an overall higher count. Among the globular proteins for which gnomAD missense mutations were mapped and analyzed (Figure S2A), 44 are observed to have this protein kinase domain. However, this number accounts for only 1.75% of the dataset (Figure S2A) due to its large size. In the much smaller IDR-partner and interacting IDR datasets (Figure S2D–I), ligand-binding domains of nuclear hormone receptors (Pfam: PF00104), PHD-finger domains (Pfam: PF00628) and core histone domains (Pfam: PF00125) stand out with high count numbers. The risk of bias is typically higher in these smaller datasets. For instance, the 11 ligand-binding domains of nuclear hormone receptors found among IDR-partners onto which SwissVar mutations were mapped (Figure S2F) make up nearly 12% of the dataset, potentially skewing the results of our enrichment analysis. Therefore, to test the robustness of our findings, we removed proteins containing the domains with overall high numbers of occurrences mentioned above and repeated our enrichment analysis. Essentially negligible changes are observed for the statistically significant ORs after the removal of these proteins (Figures S3–S5). Hence, the enrichment trends reported do not appear significantly biased by any Pfam domain overrepresentation.

The set of IDRs that we analyzed is likely enriched in those that fold upon binding, which includes peptide motifs and MoRFs [26,32,47], potentially leading to a bias against more dynamic forms of IDR interactions, namely fuzzy interactions [35,68]. To assess this concern, we compared the predicted level of intrinsic disorder and residue composition between our datasets and disordered regions from FuzDB, which is a database of fuzzy protein complexes [69]. We also compared both properties with Pfam domains to get the contrast with structured domains. First, we compared the datasets using scores from Disopred, a sequence-based predictor of disordered regions (Figure S6) [70]. The Disopred scores of the globular and IDR-partner sets are both very similar to the scores of the Pfam domains, all of which exhibit low levels of predicted disorder, which is expected from independently folding domains. On the other hand, the interacting IDRs show a distribution closer to that of FuzDB protein regions, although the overall level of predicted disorder is not as high. This difference is likely due to the presence of highly dynamic regions in FuzDB. This database includes not only polymorphic binding regions that sample multiple bound conformations but also flanking and clamping regions that are functionally important but are not the primary binding regions [69,71]. Next, we evaluated the residue composition of each dataset, which shows again that the globular and IDR-partner sets are closest to the Pfam set (Figure S7). Compared to the globular set, both FuzDB and interacting IDR sets are enriched in polar and charged residues, which are common in disordered regions. However, sequences in FuzDB have more polar residues, while those in the interacting IDR set have more charged residues. In summary, the interacting IDRs we analyzed are clearly distinguished from folded globular domains, but their sequence composition also differs slightly from disordered regions involved in fuzzy interactions. Overall, this analysis suggests that our mutation enrichment findings mainly pertain to IDR interactions that involve folding upon binding.

## 4. Discussion

IDR interactions are recognized not only for their critical role in cellular communication and regulation but also for their differences in molecular properties compared to the classical globular interactions [45,47,49,72]. It is reasonable to assume that the structural properties of protein interfaces will affect the susceptibility and tolerance of interface residues to missense mutations. In this study, we report evidence that IDR interactions are just as enriched in disease-associated mutations as globular interactions, suggesting that the interface residues from both categories of interactions are equally crucial for function. Most remarkable is the strong enrichment at the interface core of IDRs for disease mutations and its depletion in neutral ones. These trends are likely the consequence of the functional roles of the IDR interactions, which are often transient and specific interactions involved in signaling and regulation [31]. The abundance of some proteins with long IDRs is under tight cellular control [73], which would imply a high sensitivity to changes in binding affinity as well. Furthermore, some IDRs are involved in promiscuous interactions, both by flexibly binding multiple partners and by binding to globular proteins that have multiple partners (i.e., one-to-many and many-to-one interactions, respectively) [74,75]. Fewer neutral mutations and stronger evolutionary conservation were observed in residues that interact with multiple protein partners, which was postulated to be the result of additive constraints from multiple interactions [76].

The SwissVar SNV dataset enables the most direct interpretation due to the connection between these mutations and diseases. The enrichment patterns of SwissVar missense mutations indicate that both interacting IDRs and IDR-partner interfaces have residues that are critical for mediating interactions and, if mutated, lead to diseases. For IDR-partners, this interpretation is consistent with certain features we have previously found enriched at their interfaces, such as high rigidity, hydrophobicity, and conservation, which are all features associated with residues important for binding [49]. Concerning the interacting IDRs, which are inherently more dynamic, the enriched mutations are likely affecting highly conserved and often hydrophobic hotspot residues, which are key determinants of interaction affinity and are often part of conserved motifs, e.g., an SH3-binding motif [44,45,77]. Alternatively, disease-causing mutations in interacting IDRs may modulate the sampling of nascent, transient secondary structures in the unbound state. If these secondary structures are involved in binding and are present in the bound complex, changes in their sampling may alter the binding affinity [28,43]. This idea is exemplified by mutations in p53 that alter its residual helical structure and, consequently, change its affinity to MDM2 [31,78].

Similar to SwissVar SNVs, somatic cancer-associated mutations have also been found enriched at structural regions [63], but they are known to have a greater tendency to localize to the protein surface and interface regions [11]. These trends are reaffirmed in our COSMIC SNV globular interaction dataset, despite overall weaker enrichments. It is interesting to note that the rim of globular interactions is particularly enriched in somatic cancer-associated mutations. Preferential localization to the rim regions, which consists of polar and charged solvent-exposed residues, is only observed in the COSMIC dataset for the globular interactions. However, this observation is consistent with the previously reported tendency for cancer mutations to disrupt PPIs through substituting charged residues and perturbing the electrostatic component of binding affinities [11,79]. The most exciting finding of the analysis with the entire COSMIC SNV data is that the cores of interacting IDRs have the highest, statistically significant ORs. Many studies have demonstrated positive correlations between cancer and proteins harboring IDRs [41,44,53,80]. Furthermore, IDRs are enriched in sites of post-translational modifications such as phosphorylation, which are proposed to be prominent targets of cancer mutations [11,33,81]. However, a previous study had found globular domains more enriched in cancer-associated mutations than predicted interacting IDRs [41], and others have noted that cancer mutations are overrepresented within highly modular protein hubs, which incidentally tend to contain IDRs [41]. A good example of a protein with large segments of IDRs but has many more cancer-associated mutations within its globular domains is p53, which is the most frequently mutated protein in human cancers [41,82]. Therefore, the broadly-defined IDRs are likely depleted of cancer mutations compared to globular regions [41], but our results reveal the hidden enrichment within the more precise structurally-defined IDR interface regions, particularly their cores, which emphasizes the importance of detailed structural information in enrichment analyses.

Notably, only small subsets of the numerous proteins that are mutated in tumor cells are verified as oncoproteins and tumor suppressors that drive oncogenesis, which is why we further analyzed oncoproteins and tumor suppressors. Oncoproteins and tumor suppressors contribute to oncogenesis through diverging mechanisms, so, unsurprisingly, their mutation localization patterns are correspondingly different. Tumor suppressors are often deactivated through destabilizing and truncating mutations [12,13,83]. The localization patterns of COSMIC mutations in globular and IDR-partner tumor suppressors suggests that disruption of PPIs is also a deactivating mechanism. In contrast, the generally activating cancer-associated mutations in oncoproteins tend to be less destabilizing and more site-specific [79], which is reflected in the higher ORs in the protein surface and interface rim regions of the globular interaction set. Interestingly, the interface regions of IDR-partner oncoproteins have no statistically significant enrichment in cancer-associated mutations, which contrast the finding for globular oncoproteins. This finding suggests that some IDR-partner interfaces may be more robust to mutations, but this may also be the result of sparse data coverage. The lack of data also does not allow for an unambiguous interpretation of the mutation enrichments for interacting IDRs in tumor suppressors and oncoproteins. Overall, the analyses of the full COSMIC data reveal that IDR interaction interfaces are highly enriched in somatic cancer missense mutations, while those in tumor suppressors and oncoproteins exhibit intriguing differences compared to globular interfaces.

In contrast to the pathogenic disease-associated mutations, missense mutations that result from SNVs present in the general population (gnomAD) are assumed to be mostly benign, and as such, should be scarce in functionally critical regions. Buried and core regions in IDR-partners exhibit depletion levels of the entirety of gnomAD SNVs that surpasses the globular set, highlighting their functional importance. Interestingly, although the core region of interacting IDRs is also significantly depleted of gnomAD SNVs that cause missense mutations, the rim of interacting IDRs has a statistically significant enrichment of these variants, which suggests an overrepresentation of neutral and novel variants at this region. While interface rim residues also contribute to binding, there is a broader trend of enrichment of gnomAD SNVs within IDRs in general, which can be attributed to the weaker structural constraint compared to globular protein folds but is also proposed to be influenced by the higher mutation rate in the encoding genes [41,65]. In essence, the statistically robust results for all gnomAD SNVs that cause missense mutations reveal that the core regions of both IDR-partners and interacting IDRs are depleted of these variants but that the other areas of interacting IDRs are certainly more tolerant to these SNVs present in the general population.

Although individual gnomAD SNVs generally do not cause disease, one study suggested that 70% of rare mutations are mildly deleterious [23], and proteins enriched in rare mutations have been suggested to have stronger associations with diseases [84]. Consistent with this idea is our observation that rare SNVs have a much-subdued depletion pattern compared to the entire gnomAD dataset. In other words, we found a higher proportion of rare SNVs localizing to the functional regions of globular, IDR-partner, and IDR interaction structures (Figure 4B). In addition, novel mutations from rapid population growth likely contribute to the more uniform distribution of rare SNVs. By contrast, high-frequency SNVs are considered to be benign due to their common presence in the population and thus are more indicative of the tolerance to amino acid variation. Concordantly, we observed particularly strong depletion of high-frequency SNVs at the buried regions of globular proteins and IDR-partners, where substitutions would likely have the most damaging effects. Notably, the interface core of the globular interaction dataset is less depleted in high-frequency SNVs relative to the buried region. More strikingly, the IDR-partner core has a much higher OR for high-frequency SNVs. While the cores of the globular and IDR-partner sets contain many SwissVar disease-associated mutations, which are probably localizing to hotspot residues, we propose that some cores could at the same time accommodate mutations that result in disruptions mild enough to escape purifying selection and that this phenomenon is particularly relevant to the IDR-partner interfaces (see below). While most high-frequency SNVs likely arise from weak purifying selection, some may be driven by positive and balancing selection [20,23]. Indeed, sustaining a high frequency in the population is likely conditional on a positive molecular phenotype for some of these SNVs. Thus, the high proportion of such missense mutations within the IDR-partner core raises the possibility that some of these mutations could provide selective advantages or contribute to the genetic diversity in the population, which is important for evolutionary adaptation [19,85,86].

The combined observations from the different mutation categories reinforce the view that IDR-interactions are critical to human cellular functions and thus are susceptible to disease-causing missense mutations, but it also reveals a contrast between the interacting IDR and IDR-partners that needed to be addressed. While the cores of interacting IDRs demonstrate intolerance to mutations across the datasets, IDR-partners showed relatively weak depletion of high-frequency SNVs and weak enrichment of COSMIC SNVs within oncoproteins. The difference between IDR and IDR-partner interface residues is intriguing because their partnerships suggest shared functional constraints. One possible explanation for the observed difference is based on the participation of IDRs in promiscuous (i.e., one-to-many) interactions [74], which could mean more significant constraints for residues with multiple functions. However, the IDR-partners can also play the role of the promiscuous binder (i.e., many-to-one interactions) [74,87]. An alternative explanation is based on the molecular structure of the IDR interaction interfaces. We and others have previously noted that interface residues in the interacting IDR, especially the core residues, make greater individual contributions to binding than the interface residues in the IDR-partner [45,46,88]. Many IDRs interact using short motifs composed of relatively few residues. These few residues in the interacting IDR core, which are often hydrophobic and transition from being highly solvent-exposed in the unbound state to being buried upon binding, contribute more to interaction surfaces than residues on the IDR-partner side [46,47]. This difference between interacting IDR and IDR-partner interfaces may explain why both contain residues (probably hotspots) that, if mutated, lead to diseases, but that the relatively large IDR-partner interfaces also appear to accommodate other residues that may be more tolerant to variance. Proteins can evolve new functions through accumulating mutations, a process that is especially prominent in dynamic protein regions [65,85], so the IDR-partners’ apparent higher tolerance for certain mutations may have a significant role in evolutionary adaption.

Nonetheless, we must mention some limitations of our study. While the use of experimentally determined structures provides crucial data for the determination of core, rim, surface, and buried regions, this approach may bias our findings to less dynamic complexes. While NMR experiments contributed a significant number of IDR complex structures, many structures were determined through X-ray crystallography experiments, which limits our dataset to IDRs that fold upon binding. Consequently, an increasingly recognized class of complexes that exhibit conformational heterogeneity in their bound state, known as fuzzy complexes, may be underrepresented [71]. Because a focal finding of this study is the importance of the core residues in interacting IDRs for function and disease, the method of identifying the core residues from IDR interaction structures is central to our investigation. Thus, fuzzy complexes in which key binding residues remain dynamic in their bound states may require a different approach to investigate mutation enrichment patterns in the future. It also needs to be stressed that some of the differences that we see between IDRs and IDR-partners are observed in sets with small numbers of data points. Therefore, additional analyses with larger datasets are required in the future to confirm the observed differences in SNV enrichments between IDRs and IDR-partners.

## 5. Conclusions

Investigating the enrichment of different categories of missense mutations within IDR interaction structures revealed several notable characteristics. Although limited in availability, IDR complex structures are crucial for precisely identifying the core residues of the interacting IDRs. While the categorization of PPI interface residues into core and rim is a well-established practice for globular proteins, the same is often not done for studying interacting IDRs. Once we identified the core residues, we more clearly observed that interacting IDR core residues are significantly enriched in SwissVar and COSMIC missense mutations as well as being depleted in gnomAD SNVs that cause missense mutations. These results suggest that interacting IDR core residues are highly intolerant to missense mutations, which support the view that the disruption of IDR interactions, and thus the cellular functions that they perform, is a common mechanism underlying many diseases. Interestingly, the trends that we observed suggest an asymmetry across the IDR interaction interface in the enrichment of certain missense mutation types. However, future analyses with more variant data will be required to confirm differences in variant enrichment between interacting IDRs and IDR-partners. In any case, the growing availability of protein structure and sequence data has enabled us to recognize important distinctions between globular and IDR-mediated interactions, and this trend continues to accelerate. Accounting for such differences will contribute to the understanding and prediction of the effects of missense mutations on disease susceptibility, which is a critical aspect of personalized medicine.

## Supplementary Materials

The following are available online at https://www.mdpi.com/2218-273X/10/8/1097/s1, Figure S1: Flow chart of structural data processing procedure with sequence and structure tabulation. Figure S2: Pfam occurrences in mutation-mapped datasets. Figure S3: Odds ratios of SwissVar SNVs excluding selected frequent Pfam domains. Figure S4: Odds ratios of COSMIC SNVs excluding selected frequent Pfam domains. Figure S5: Odds ratios of gnomAD SNVs excluding selected frequent Pfam domains. Figure S6: Box plot of Disopred disorder prediction scores. Figure S7: Residue composition difference between the respective dataset and the globular dataset. Table S1: Tabulated residues and mutations used in odds ratios calculations. Table S2: Odds ratios and statistics.
