# Supplementary material for: Protein–Protein Interactions Mediated by Intrinsically Disordered Protein Regions Are Enriched in Missense Mutations
Source: Biomolecules. 2020 Jul 24;10(8):1097. doi: 10.3390/biom10081097 (PMC7463635; doi:10.3390/biom10081097)
Supplement: Supplementary file 1 [file biomolecules-10-01097-s001.zip › Supplementary_Materials.pdf]

**Protein–Protein Interactions Mediated by Intrinsically Disordered Protein Regions Are Enriched in Missense Mutations. Biomolecules 2020**

**Supplementary Material**

**Supplementary Figures S1-S7**

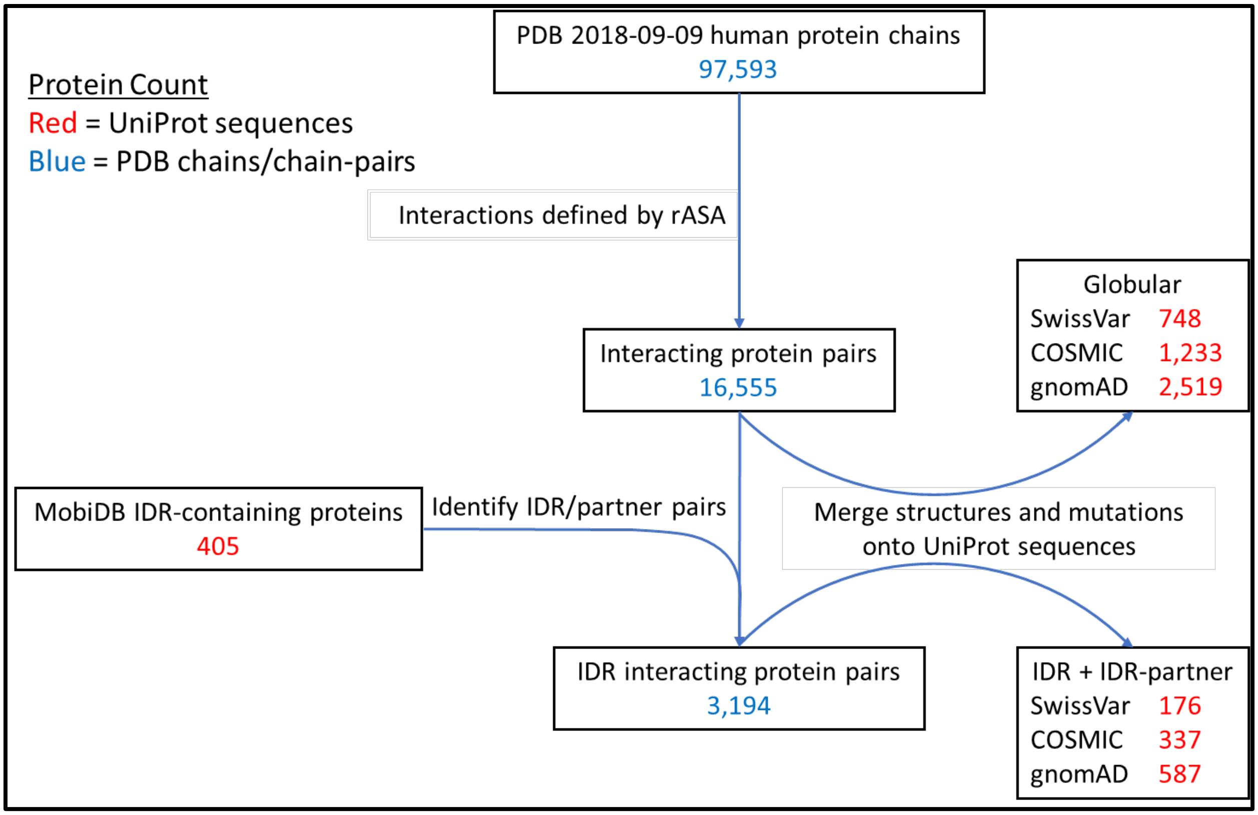


**Supplementary Figure S1.** Flow chart of structural data processing procedure with sequence and structure tabulation. Relative solvent accessible surface areas (rASAs) were calculated on protein chain pairs from the PDB to identify interacting protein pairs and define structural regions, which are subsequently mapped onto UniProt sequences and merged with mutation data (see Methods for details). The IDR interactions were identified by mapping IDRs defined in MobiDB onto interacting protein pairs. Blue numbers indicate PDB chains and structures, which include redundant or overlapping structures. Red numbers represent UniProt sequences, and each sequence contains one or more regions with structural data. For example, one UniProt sequence from the 587 sequences indicated in the lower right corner could contain multiple IDR-partners and interacting IDRs.

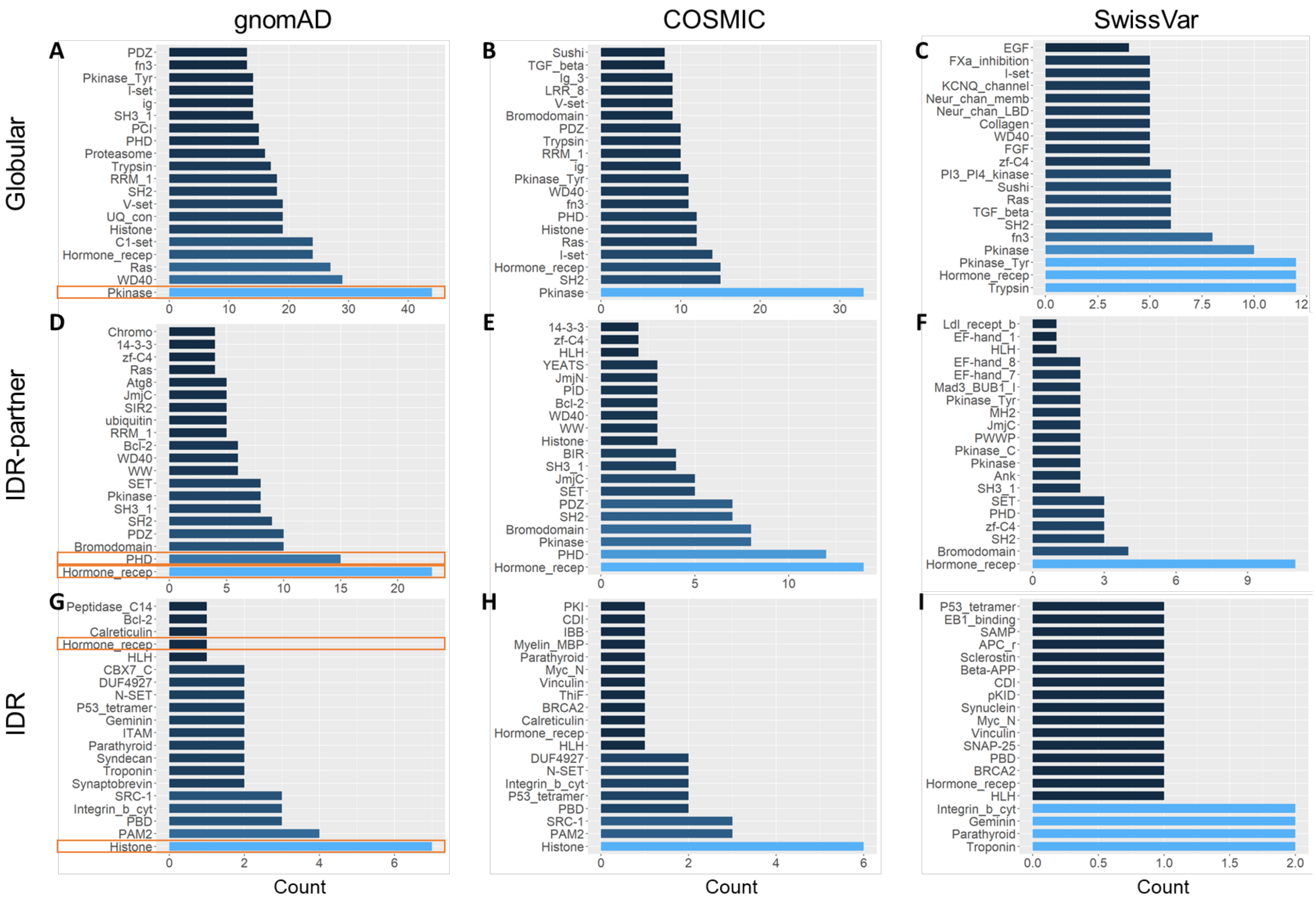


**Supplementary Figure S2.** Pfam occurrences in mutation-mapped datasets. For each dataset, we tabulated the Pfam domains that overlap with the respective structured regions under investigation. The X-axis is the count of the proteins containing each Pfam domain. The Y-axis shows the

name of each domain and is ordered based on frequency. The analysis included all types of Pfam entries. The orange boxes highlight the selected outlying domains which were chosen based on their frequency in the globular (A), IDR-partner (D), and interacting IDR (G) gnomAD datasets. For the “robustness” analysis shown in Figures S3, S4, and S5, we removed the protein kinase domains (Pfam: PF00069) in the globular set, and the ligand-binding domains of nuclear hormone receptors (Pfam: PF00104), PHD-finger domains (Pfam: PF00628), and core histone domains (Pfam: PF00125) in the IDR-partner and IDR sets.

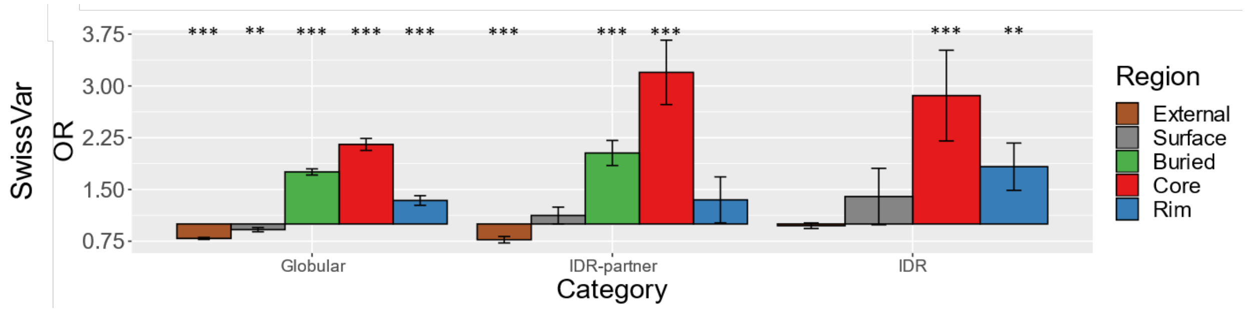


**Supplementary Figure S3.** Odds ratios of SwissVar SNVs excluding selected frequent Pfam domains. Proteins in which the structured regions overlap with the most frequent Pfam domains were removed for the calculation of odds ratios. See Supplementary Figure S2 and Figure 2 for more details.

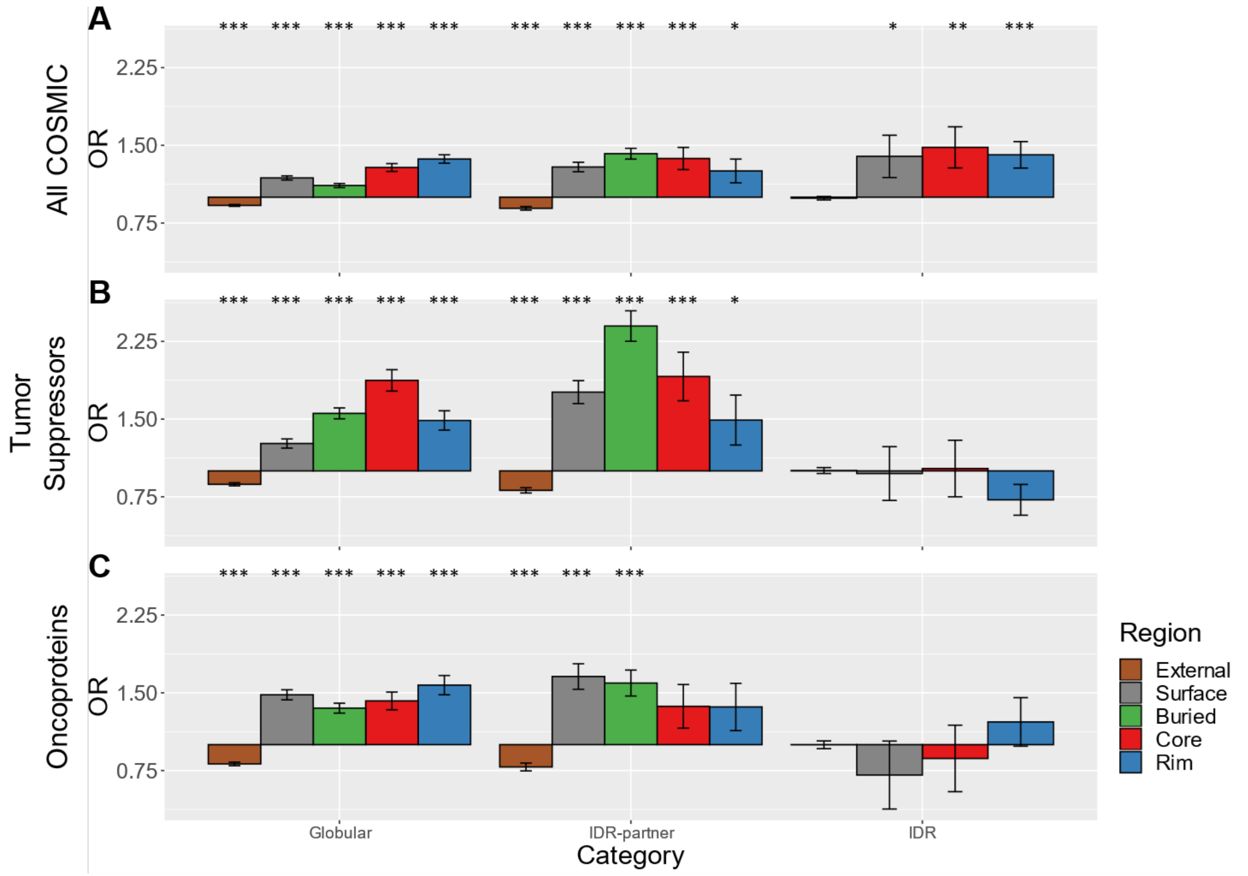


**Supplementary Figure S4.** Odds ratios of COSMIC SNVs excluding selected frequent Pfam domains. Proteins in which the structured regions overlap with the most frequent Pfam domains were removed for the calculation of odds ratios. See Supplementary Figure S2 and Figure 3 for more details.

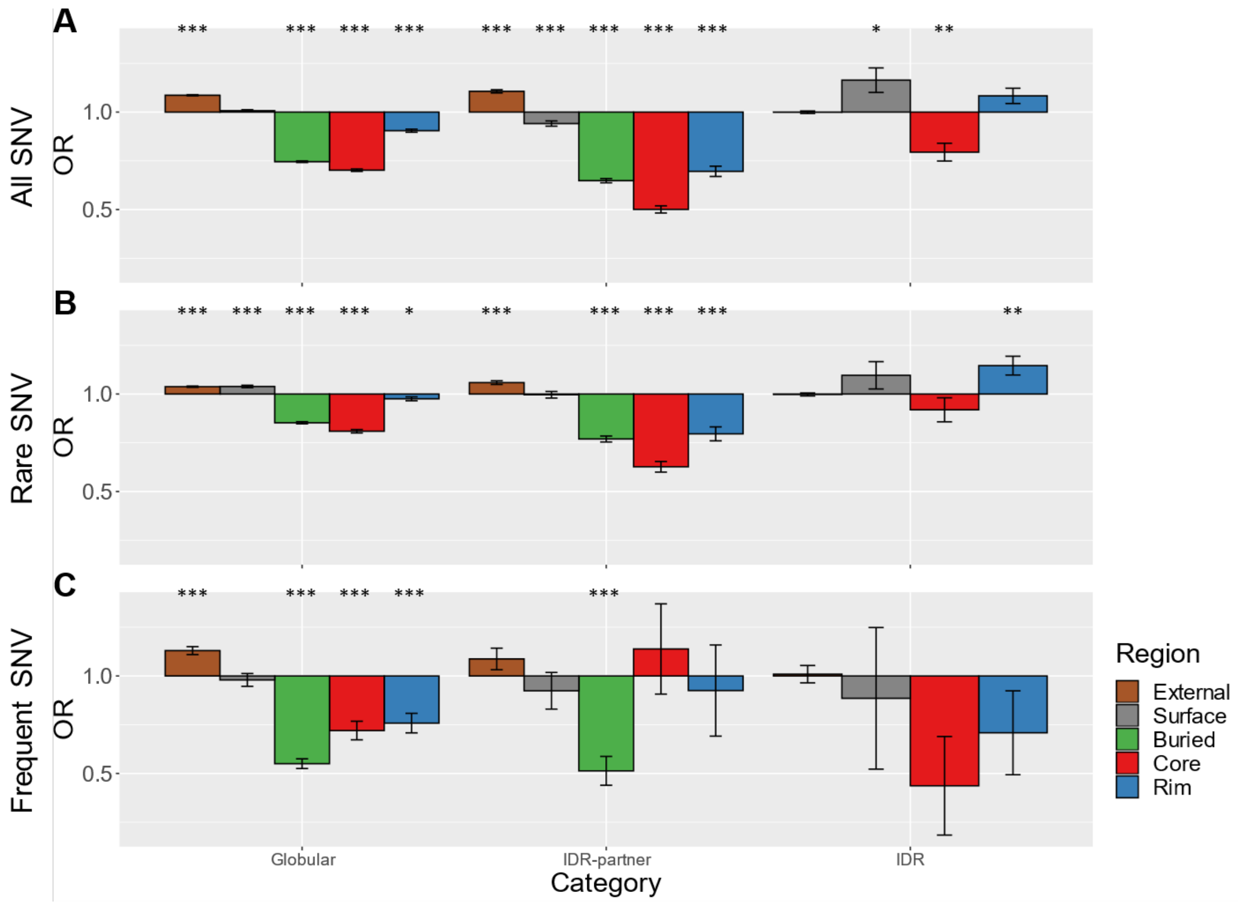


**Supplementary Figure S5.** Odds ratios of gnomAD SNVs excluding selected frequent Pfam domains. Proteins in which the structured regions overlap with the most frequent Pfam domains were removed for the calculation of odds ratios. See Supplementary Figure S2 and Figure 4 for more details.

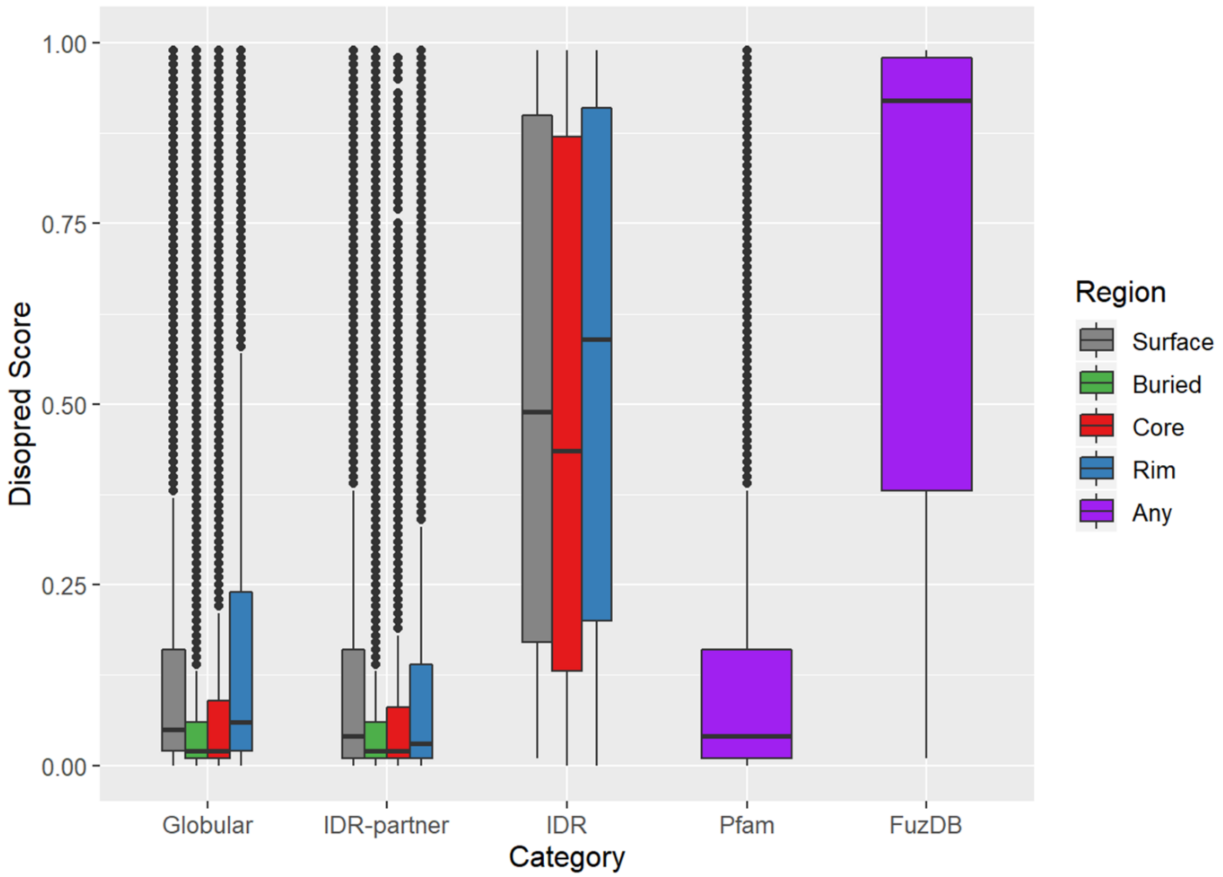


**Supplementary Figure S6.** Box plot of Disopred disorder prediction scores. For the annotated sequence regions of each dataset, we plotted the per-residue disorder prediction scores calculated using Disopred3. The disorder scores range from 0 to 1, representing order and disorder, respectively. The globular, IDR-partner, and IDR datasets were introduced in the Methods section of the main text. The Pfam dataset consists of sequences of domains from the Pfam database. The FuzDB dataset consists of disordered regions curated in FuzDB that we extracted from MobiDB. For Pfam, the region "Any" includes all types of Pfam entries mapped on human UniProt sequences, but most Pfam entries are domains. For FuzDB, the region "Any" includes all topological classes of disordered regions mapped on human UniProt sequences, including polymorphic, clamp, flanking, and random disordered regions. Notably, FuzDB dataset only includes 41 human proteins.

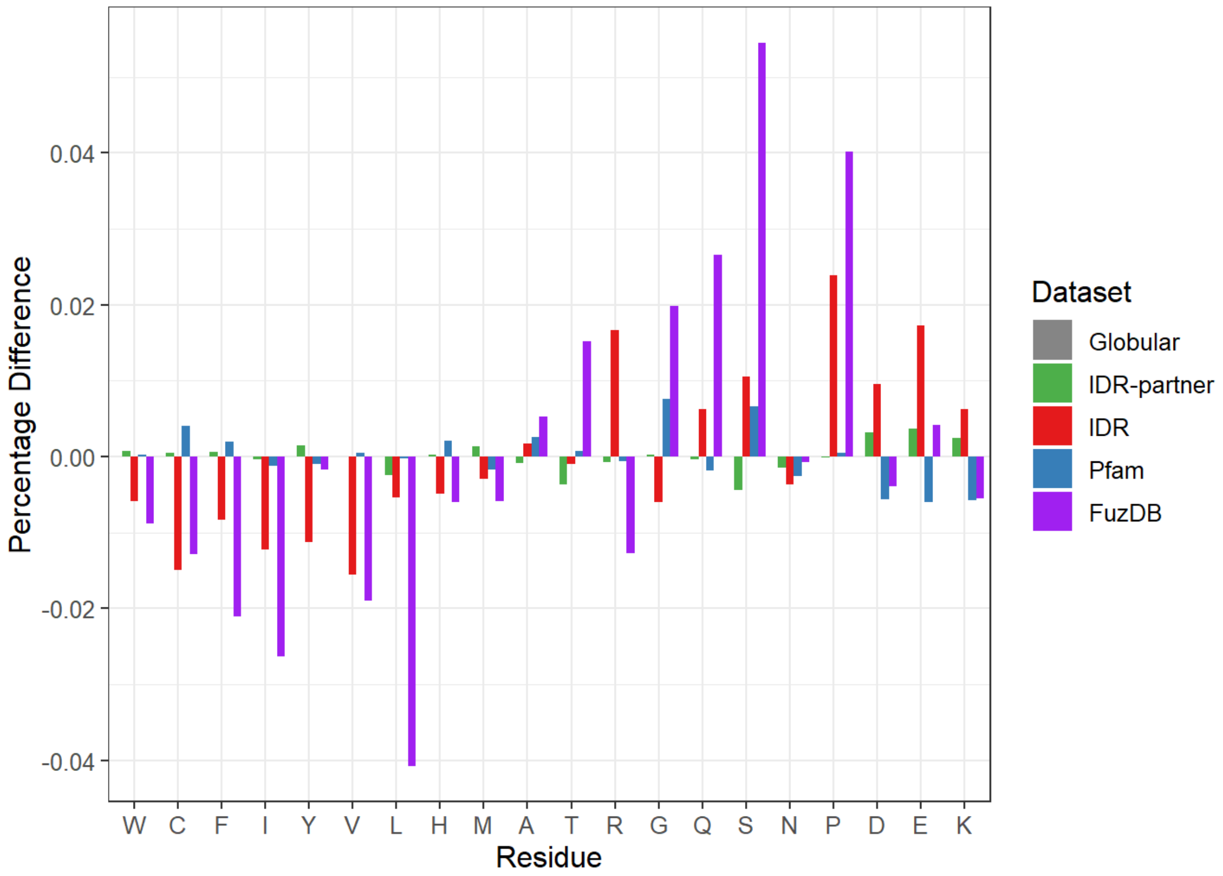


**Supplementary Figure S7.** Residue composition difference between the respective dataset and the globular dataset. The number of each residue type was normalized by the total number of residues to calculate the percentage composition of each dataset. Subsequently, we subtracted the percentage composition by the values from the globular dataset to get the percentage difference (Y-axis). Therefore, positive values indicate residue types that are enriched relative to the globular dataset, while negative values indicate depletion. The residue types on the X-axis are ordered based on their ranking in protein flexibility [1], with residues associated with higher disorder propensity on the right side.

**References:**


1. Vihinen, M.; Torkkila, E.; Riikonen, P. Accuracy of protein flexibility predictions. *Proteins* **1994**, *19*, 141–149.
